# Clinical trials: The mathematics of falling vaccine efficacy with rising disease incidence

**DOI:** 10.1016/j.vaccine.2016.04.065

**Published:** 2016-06-08

**Authors:** M. Gabriela M. Gomes, Stephen B. Gordon, David G. Lalloo

**Affiliations:** a Liverpool School of Tropical Medicine, United Kingdom; b CIBIO-InBIO, Centro de Investigação em Biodiversidade e Recursos Genéticos, Universidade do Porto, Portugal; c Instituto de Matemática e Estatística, Universidade de São Paulo, Brazil; d Malawi Liverpool Wellcome Trust Programme of Clinical Tropical Research, Blantyre, Malawi

Reports of unexplained discrepancies in the efficacy of vaccines, as estimated from randomised controlled trials in different parts of the world, are commonplace in the literature [1], [2], [3], [4]. Moreover, there is a consistent trend for lower vaccine efficacy when measured in settings where the disease of interest has a higher incidence, leading to questions about the appropriateness of pooled estimates. Here, we examine the mathematical basis for such trends and propose a measure of efficacy that is valid across settings. The approach relies on fitting mechanistic models, which specify pathogen exposures and host responses, to global vaccine trial data stratified by local disease incidence. Such models enable the estimation of vaccine protection per exposure to the pathogen. A strategy to estimate per-exposure vaccine efficacy will enable more accurate estimates of vaccine efficacy across a range of disease incidence [5].

## 1 Minimal model for a clinical trial

Any analysis of a vaccine trial must compare the incidence of disease in two groups of the population that are differentiated by whether or not they have received the vaccine, here named the vaccine group and the control group. Following Smith et al. [6] and Halloran et al. [7] for leaky vaccines, we denote by *r*_c_ = *λ* the *per capita* rate of infection of unvaccinated individuals (force of infection) and by *r*_v_ = *σλ* the reduced rate of infection in vaccinated subjects. The efficacy of the vaccine is then represented by 1 − *r*_v_/*r*_c_ = 1 − *σ*. As noted by Smith et al. [6], this measure based on rate ratios is independent of time while a measure based on risk ratios declines over time from the beginning of the trial due to depletion of individuals at risk. Here, however, we are more interested in stressing independence on the intensity of transmission that governs the incidence of disease, and focus on rate ratios while Margheri et al. [9] elaborates on both rate and risk measures. Despite these theoretical notions of independence, we encounter a consistent trend of decreasing vaccine efficacy with increasing force of infection when estimated by conventional randomized controlled trials.

## 2 Relaxing the model and adjusting the data

**Fig. 1:**
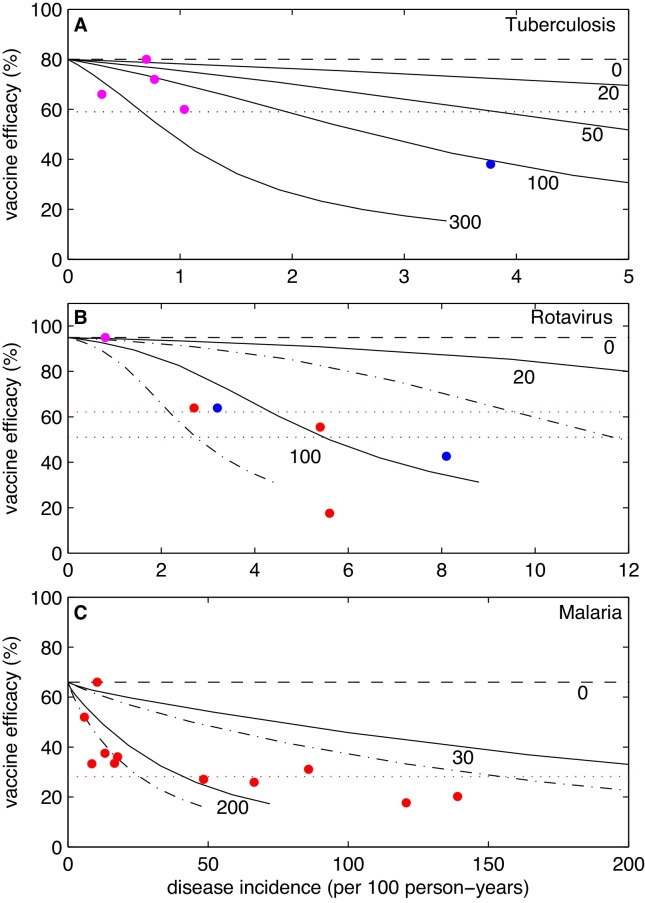
Vaccine efficacy on disease incidence gradients. (a) Tuberculosis [1]. (b) Rotavirus [2], [3], [4]. (c) Malaria [10]. Solid curves are generated with the formula 1 − *r*_v_/*r*_c_, where *r*_c_ and *r*_v_ represent the incidence of infection in the control and vaccine groups, over 1 year, and the labels indicate the variance of the risk distribution, *q*(*x*), in the absence of vaccination. Dash-dotted lines are the same measures calculated over longer follow-up (2 years in the case of rotavirus, 4 years in the case of malaria). Data points come from settings where a trial has been conducted to estimate vaccine efficacy (Tables S1, S2, and S3), and dotted lines represent average estimates by continent (America (magenta), Asia (blue), Africa (red)) taken from the originally published trial analyses.

We extracted data on vaccine protection for tuberculosis (bacille Calmette-Guérin, BCG [1]), rotavirus (pentavalent rotavirus vaccine, RotaTeq [2], [3], [4]), and malaria (RTS,S [10]), from systematic reviews and recent multicenter clinical trials. These are plotted in Fig. 1 to illustrate our argument (see appendix for tables). The dashed lines represent 1 − *σ*, which we set at the level of the highest protection estimate from any of the studies. As changing *σ* basically moves the lines up and down, it is evident that this cannot satisfactorily fit the trends for reduced vaccine protection with increased incidence seen from real life data. Based on the fact that individual risks of infection and disease are not homogeneous even in local settings, a model can be developed that accounts for heterogeneous distributions of individual risks [9], [11], resulting in instantaneous rates written as *r*_c_(*t*) = ∫ *λxe*^−*λxt*^*q*(*x*)d*x*/∫ *e*^−*λxt*^*q*(*x*)d*x* and *r*_v_(*t*) = ∫ *σλx* *e*^−*σλxt*^*q*(*x*)d*x*/∫ *e*^−*σλxt*^*q*(*x*)d*x*, where *q*(*x*) is the probability density function of individual risk. Heterogeneity induces a cohort selection effect [12], [13] whereby individuals at higher risk are infected first and leave behind less susceptible subjects. This results in disease rates that decrease over time [14], an output that can be tested against time-to-event data. This effect is more pronounced in the control group as individuals within it experience higher rates of infection overall. Consequently, the ratio of disease rates in vaccinated over control groups increases, and vaccine efficacy, as measured by simple rate ratios, decreases as the trial progresses. Finally, the magnitude of this effect increases with the intensity of transmission.

To illustrate the effect just described, we use a gamma distribution to represent heterogeneity in individual disease risk and generate the set of curves added to the plots in Fig. 1. Solid curves, corresponding to 1-year follow-up, are labeled by the variance of the respective gamma distribution, which can be adjusted for best fitting the data (for the malaria trial we used a modified model to account for repeated infections [9]). In the case of rotavirus and malaria vaccines, we refer to recent trials, which conform with standardized multicenter designs, a highly desirable attribute for global analyses such as this. The original publications report follow-up periods of 2 years for rotavirus [2], [3], [4] and 4 years for malaria [10], and these prolonged durations are represented by the dash-dotted curves. In any case, the trend of falling vaccine efficacy with rising disease incidence can be explained by a simple selection mechanism, which is not accounted for in standard trial analyses. The measured variations in vaccine effects can be reproduced when a plausible model is fitted to global data, stratified by country or some other unit that enables the specification of disease occurrence ratios by incidence. The approach involves fitting a curve to estimate two parameters: the risk ratio of vaccine over control groups, *σ*; and the variance of the risk distribution in control groups, *q*(*x*). Vaccine efficacy is then defined as 1 − *σ*, representing, effectively, a measure of efficacy per unit of exposure, which has a clear interpretation and can be used to parameterize predictive models of vaccine protection in different incidence settings [15].

The procedure can be refined as much as detailed attributes of trial participants and pathogen types are collected and modeled, although this requires access to original individual data, rather than the aggregate measures that are usually published. The field would gain substantially if individual patient data were deposited in repositories such as ImmPort or other public portals.

## 3 Observations from vaccine trials

Fig. 1 shows information retrieved from published vaccine trials. Each data point comes from a country where a trial has been conducted to estimate the efficacy of a specific vaccine (colour coded by continent). Dotted lines represent average estimates reported in the original publications: BCG against pulmonary tuberculosis [1]; RotaTeq against rotavirus gastroenteritis [2], [3], [4]; RTS,S against malaria [10]. Three points are worth highlighting. The first concerns the reporting of overall estimates taken as averages. When variation in vaccine effects is large, pooled estimates have little meaning as seen by comparing the dotted lines with the data points. The second is that a suitable mathematical model can generate a family of curves that describe the observed trends, and the parameters that identify the best fitting curve can be estimated by available statistical inference procedures [16], [17], [18]. The third is that the efficacy parameter obtained by this procedure approximates the vaccine effect per unit of exposure [5].

## 4 Improving estimations from trials

There is overwhelming evidence that vaccine efficacy, as commonly estimated in randomized controlled trials, decreases with increasing disease incidence, but these observations can be reconciled by making the analysis less rigid. We show that a plausible model can reproduce the incidence-dependent observations when adequately parameterized and give a vaccine efficacy measurement that is valid for all incidences. This universal efficacy quantity can be derived, by fitting the model to vaccine trial data over a gradient of incidences. Adjusting models to global data requires flexible distributions of individual disease risk to be implemented, which can also be estimated in this process [19], [20]. For illustration, we have chosen three vaccines that have undergone randomized controlled trials throughout the globe (BCG against pulmonary tuberculosis [1], RotaTeq against rotavirus gastroenteritis [2], [3], [4] and RTS,S against malaria [10]), but the procedure is applicable more generally to vaccines and other interventions against not only infectious diseases, but also non-communicable disorders provided that information is available on exposure intensity to a specific risk factor.

We hope to have laid the basis for further research on this important topic where methodological development can be as elaborate as the detail in available data allows. Per-exposure efficacy can be estimated by fitting models to data from multicenter clinical trials, ideally with study sites selected as to cover the largest possible range of transmission intensities.

While here we focus on the mathematical basis for the reported trends, large studies are in progress to unravel biological determinants of immune response to vaccines in a setting-specific manner. The two viewpoints are not exclusive but complementary, with the possibility of each only partially explaining the observations. Further studies are needed to identify how much of the trend can be explained by the mathematical argument offered here. Dose−response experimental challenge systems are ideally suited to this objective [8], and the recent attention devoted to the establishment of safe protocols applicable to human volunteers offers a unique and exciting opportunity [21].
