# Supplementary material for: Clinical trials: The mathematics of falling vaccine efficacy with rising disease incidence
Source: Vaccine. 2016 Jun 8;34(27):3007–9. doi: 10.1016/j.vaccine.2016.04.065 (PMC5087849; doi:10.1016/j.vaccine.2016.04.065)
Supplement: Supplementary file 1 [file mmc1.pdf]

Data supplement

Table S1: Bacille Calmette-Guérin vaccine (BCG) protection against pulmonary tuberculosis used in Figure 1A. Neonatal vaccination data from Mangtani et al 2014 [1], structured by study site.

Site, Country	Incidence density (per 100 person-years at risk)	Vaccine efficacy % (95% CI)
Saskatchewan, Canada	0·7	80 (31, 94)
Chicago Cook Country Hospital, USA	0·3	66 (40, 81)
Turtle and Rosebud, USA	1·0	60 (-25, 87)
Chicago tuberculous households, USA	0·8	72 (-2, 92)
Bombay, India	3·8	38 (-7, 65)
Overall	---	59 (42, 71)

Table S2: Pentavalent rotavirus protection (RotaTex) efficacy used in Figure 1B. Infant vaccination data from Vesikari et al 2007 [2], Armah et al 2010 [3], Zaman et al 2010 [4], structured by country.

Country	Incidence density (per 100 person-years at risk)	Vaccine efficacy % (95% CI)
USA[2]	0·8	94·9 (84·0, 98·9)
Ghana[3]	5·4	55·5 (28·0, 71·1)
Kenya[3]	2·7	83·4 (-5·9, 89·8)
Mali[3]	5·6	17·6 (-22·9, 45·0)
Africa overall[3]	---	39·3 (19·1, 54·7)
Bangladesh[4]	8·1	42·7 (10·4, 63·9)
Vietnam[4]	3·2	63·9 (7·6, 90·9)
Asia overall[4]	---	48·3 (22·3, 66·1)

Table S3: RTS,S/AS01 vaccine protection against clinical malaria used in Figure S2. African children (intention-to-treat population in the 5-17 age category) vaccination data (primary schedule without booster) from RTS,S Clinical Trials Partnership 2015 [5], structured by study site.

Site, Country	Incidence density* (per 100 person-years at risk)	Vaccine efficacy % (95% CI)
Kilifi, Kenya	10·4	66·0 (37·5, 81·5)
Korogwe, Tanzania	5·9	52·0 (26·2, 68·8)
Manhiça, Mozambique	8·5	33·3 (7·1, 52·1)
Lambaréné, Gabon	17·7	36·1 (10·8, 54·1)
Bagamoyo, Tanzania	13·2	37·5 (13·5, 54·9)
Lilongwe, Malawi	16·6	33·5 (8·2, 51·8)
Agogo, Ghana	85·8	31·1 (13·3, 45·2)
Kombewa, Kenya	48·4	27·1 (12·9, 38·9)
Kintampo, Ghana	66·4	25·9 (15·0, 35·4)
Nianoro, Burkina Faso	120·7	17·7 (7·0, 27·2)
Siaya, Kenya	139·0	20·2 (7·4, 31·3)
Africa Overall	---	28·2 (23·3, 32·9)

* Calculated form RTS,S Clinical Trials Partnership 2015 [5] as I/P, where I is the cumulative number of clinical malaria cases and P is person-years at risk in the respective group.

References

1. Mangtani P, Abubakar I, Ariti C, et al. Protection by BCG vaccine against tuberculosis: a systematic review of randomized controlled trials. *Clin Infect Dis* 2014; 58: 470-80.
2. Vesikari T, Itzler R, Matson DO, et al. Efficacy of a pentavalent rotavirus vaccine in reducing rotavirus-associated health care utilization across three regions (11 countries). *Int J Infect Dis* 2007; 11:29-35.
3. Armah GE, Sow SO, Breiman RF, et al. Efficacy of pentavalent rotavirus vaccine against severe rotavirus gastroenteritis in infants in developing countries in sub-Saharan Africa: a randomised, double-blind, placebo-controlled trial. *Lancet* 2010; 376: 606-14.
4. Zaman K, Dang DA, Victor JC, et al. Efficacy of pentavalent rotavirus vaccine against severe rotavirus in infants in developing countries in Asia: a randomised, double-blind, placebo-controlled trial. *Lancet* 2010; 376: 615-23.
5. The RTS,S Clinical Trials Partnership. A phase 3 trial of RTS,S/AS01 malaria vaccine in African infants. *N Engl J Med* 2015; 367: 2284-95.
